# Cardiorespiratory fitness, muscle fitness, and physical activity in children with long QT syndrome: A prospective controlled study

**DOI:** 10.3389/fcvm.2022.1081106

**Published:** 2023-01-11

**Authors:** Luc Souilla, Martina Avesani, Aymeric Boisson, Anne Requirand, Stefan Matecki, Marie Vincenti, Oscar Werner, Gregoire De La Villeon, Victor Pommier, Jean-Luc Pasquie, Sophie Guillaumont, Pascal Amedro

**Affiliations:** ^1^Department of Paediatric and Congenital Cardiology, M3C Regional Reference Centre, University Hospital, Montpellier, France; ^2^PhyMedExp, Univ Montpellier, INSERM, French National Centre for Scientific Research (CNRS), Montpellier, France; ^3^Department of Paediatric and Adult Congenital Cardiology, M3C National Reference Centre, Bordeaux University Hospital, Pessac, France; ^4^Department of Physiology, University Hospital of Montpellier, Montpellier, France; ^5^Paediatric Cardiology and Rehabilitation Unit, Saint-Pierre Institute, Palavas-Les-Flots, France; ^6^Inserm, U1045, Institut Hospitalo-Universitaire (IHU) Liryc, Bordeaux Cardio-Thoracic Research Centre, Electrophysiology and Heart Modelling Institute, University of Bordeaux, Pessac, France

**Keywords:** pediatrics, long QT syndrome, inherited cardiac arrythmia, cardiorespiratory fitness, muscle fitness, physical activity

## Abstract

**Background:**

In children with congenital long QT syndrome (LQTS), the risk of arrhythmic events during exercise commonly makes it difficult to balance exercise restrictions *versus* promotion of physical activity. Nevertheless, in children with LQTS, cardiorespiratory fitness, muscle fitness, and physical activity, have been scarcely explored.

**Materials and methods:**

In this prospective, controlled, cross-sectional study, 20 children with LQTS (12.7 ± 3.7 years old) and 20 healthy controls (11.9 ± 2.4 years old) were enrolled. All participants underwent a cardiopulmonary exercise test, a muscular architecture ultrasound assessment, (cross-sectional area on right rectus femoris and pennation angle), a handgrip muscular strength evaluation, and a standing long broad jump test. The level of physical activity was determined using with a waist-worn tri-axial accelerometer (Actigraph GT3X).

**Results:**

Peak oxygen uptake (VO_2peak_) and ventilatory anaerobic threshold (VAT) were lower in children with LQTS than in healthy controls (33.9 ± 6.2 mL/Kg/min *vs.* 40.1 ± 6.6 mL/Kg/min, *P* = 0.010; 23.8 ± 5.1 mL/Kg/min *vs.* 28.8 ± 5.5 mL/Kg/min, *P* = 0.007, respectively). Children with LQTS had lower standing long broad jump distance (119.5 ± 33.2 cm *vs.* 147.3 ± 36.1 cm, *P* = 0.02) and pennation angle (12.2 ± 2.4° *vs.* 14.3 ± 2.8°, *P* = 0.02). No differences in terms of moderate-to-vigorous physical activity were observed (36.9 ± 12.9 min/day *vs.* 41.5 ± 18.7 min/day, *P* = 0.66), but nearly all children were below the WHO guidelines.

**Conclusion:**

Despite similar physical activity level, cardiorespiratory fitness and muscle fitness in children with LQTS were lower than in healthy controls. The origin of this limitation seemed to be multifactorial, involving beta-blocker induced chronotropic limitation, physical and muscle deconditioning. Cardiovascular rehabilitation could be of interest in children with LQTS with significant physical limitation.

## 1. Introduction

Habits and beliefs about physical activity in the general population are frequently not in line with the guidelines and recommendations (1). In pediatrics, according to the recent guidelines on physical activity from the World Health Organization (WHO), children aged 5 to 17 years old should perform at least 60 min of moderate-to-vigorous physical activity, daily (2, 3). Despite the physiological and psychological health benefits related to physical activity across the lifespan (4, 5), nearly 80% of children does not meet these recommendations (6). This is particularly true for children with chronic diseases, for which exercise intolerance and physical inactivity (7) contribute to increase cardiovascular risk during adulthood (8, 9). Cardiorespiratory fitness reflects children’s global health and stands as an independent predictor of all-cause mortality (10).

In the wide spectrum of pediatric chronic diseases, children with cardiac diseases are particularly affected by physical inactivity, as a consequence of their underlying cardiac condition, but also of parental overprotection and social barriers to sports practice, which negatively affect health-related quality of life (11, 12). Similarly, in children with inherited cardiac arrhythmia, such as long QT syndrome (LQTS) (13, 14), the risk of arrhythmic events during exercise commonly makes it difficult to balance exercise restrictions *versus* promotion of physical activity (15–19). However, the association between exercise and sudden cardiac death in LQTS remains unclear (20, 21).

In the past two decades, the guidelines on sports participation in inherited cardiac disorders have become progressively less restrictive (22–25) and the concept of shared-decision making, involving patients, families, and physicians, has recently emerged to promote physical activity, even in patients with LQTS (22). These guidelines are not primarily dedicated to pediatric patients, and physical fitness in children with LQTS has been scarcely explored.

In this study, we aimed to evaluate cardiorespiratory fitness, muscle fitness (strength and architecture), and physical activity, in children with LQTS, in comparison with healthy controls.

## 2. Materials and methods

### 2.1. Study design and population

This prospective controlled cross-sectional study was carried out from February to June 2021 in two pediatric cardiology tertiary care hospitals: the Pediatric and Congenital Cardiology Department of Montpellier University Hospital, France; and the Pediatric Cardiology and Rehabilitation Unit of the St-Pierre Institute in Palavas-Les-Flots, France.

Children aged between 6 and 18 years old were screened consecutively during a regular pediatric cardiology outpatient visit. Two groups were identified:

1. The LQTS group consisted of children diagnosed with congenital long QT syndrome, which is characterized by QT prolongation in repeated 12-lead electrocardiogram (ECG), and/or genetic mutation in children screened for known causal familial LQTS (26).
2. The control group consisted of children referred for a non-severe functional symptom (cardiac murmur, chest pain). These children were classified in the control group only after a comprehensive cardiac evaluation which revealed no cardiopulmonary abnormalities, including physical examination, ECG, and echocardiography. Children with any chronic disease, medical condition (cardiac, neurological, respiratory, muscular, or renal), or medical treatment and those requiring any further specialized medical consultation were not eligible.

Children with absolute contraindications for cardiopulmonary exercise test (CPET) were not eligible: fever, uncontrolled asthma, respiratory failure, acute myocarditis or pericarditis, uncontrolled arrhythmias causing symptoms or hemodynamic compromise, uncontrolled heart failure, acute pulmonary embolus or pulmonary infarction, and children with intellectual or developmental disability which impaired their ability to complete the exercise protocol.

### 2.2. Physical fitness

Two main components of physical fitness were evaluated: (1) cardiorespiratory fitness, and (2) muscle fitness (27).

#### 2.2.1. Cardiorespiratory fitness

Children from both groups underwent a CPET, using a pediatric cycle ergometer protocol adapted to children to obtain a homogeneous incremental overall duration between 8 and 12 min, as previously described by our group (28). CPET procedures in both centers were harmonized before the study started. We used the Quark CPET calibrated gas analyzer (Cosmed Srl., Pavonna di Albano, Italy). The following CPET parameters were measured: peak oxygen uptake (VO_2peak_), peak heart rate (HR_peak_) ventilatory anaerobic threshold (VAT), ventilatory efficiency (VE/VCO2 slope), oxygen pulse (VO2/HR), maximal power, respiratory exchange ratio (RER), respiratory reserve (RR). Spirometry was systematically performed before the exercise test, to measure forced expiratory volume in 1 s (FEV1), forced vital capacity (FVC), and the FEV1/FVC ratio. The CPET was considered as maximal when the following four criteria were reached: respiratory exchange ratio (RER = VCO_2_/VO_2_) ≧ 1.1, and limit of the child’s tolerance despite verbal encouragement. VO_2peak_ values were normalized as percentage of the predicted VO_2peak_. A single investigator manually calculated the VO_2peak_ and the VAT using Beaver’s method (29). VO_2peak_ and VAT values were normalized in a percentage of the predicted maximum oxygen uptake using Cooper’s pediatric reference values (30). A VO_2peak_ value below 80% and/or a VAT value below 55% of predicted VO_2peak_ were indicative of physical deconditioning, in reference to reported values in adults and children (31).

#### 2.2.2. Muscle fitness

Muscle fitness was determined by evaluating muscle architecture and muscle strength (32).

##### 2.2.2.1. Muscle architecture

**FIGURE 1:**
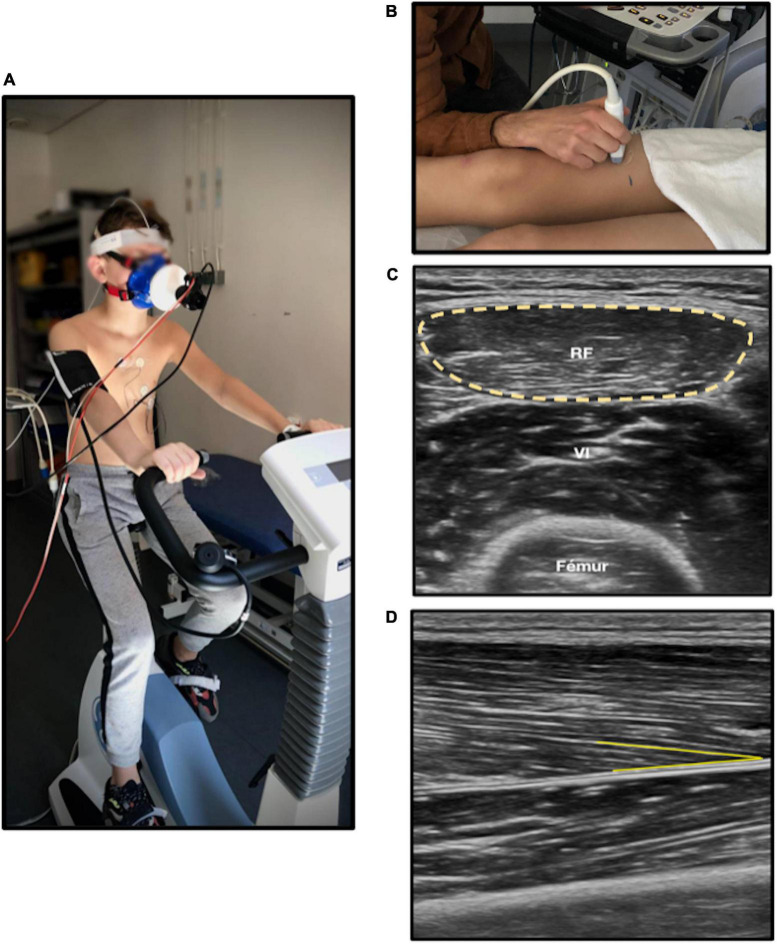
Cardiorespiratory and muscle fitness analysis. **(A)** Cardiopulmonary exercise test on ergocycle. **(B)** Muscular ultrasound on right leg. **(C)** Anatomical cross-sectional area (yellow circle) of rectus femoris measured with ultrasound. **(D)** Pennation angle (yellow line) of right vastus lateralis; RF, Rectus femoris. The chosen anatomic site was at two third of the length from iliac spine anterior superior to upper edge of the patella on right leg. The probe was put in transversal plane and longitudinal plane for cross-sectional area and pennation angle, respectively.

Muscle architecture reflects the contractile properties of muscle and is a determinant of strength capacity (33). It was evaluated by muscular ultrasound technique (34), the patient being in dorsal decubitus, with legs and arms relaxed, feet in neutral position, and arm in supination. Analyses of cross-sectional area on rectus femoris showed good feasibility and reliability (35). We measured two parameters: (1) the anatomical cross-sectional area on right rectus femoris, defined as the area of the cross-section of the muscle perpendicular to its longitudinal axis; (2) the pennation angle of vastus lateralis (Figure 1). Pennation angle was defined as the angle formed between muscular fascicles and intramuscular tendon insertion. A greater pennation angle enables more myofibrillar packing and promotes fascicle rotation during dynamic contraction strength, increasing muscle cross-sectional area and strength (36, 37). Thus, a lesser pennation angle may suggest reduced strength capacity (38). Five measures were performed for each one of the two parameters by a single operator. Minimal and maximal values were excluded, and the mean of the three remaining values was calculated. Image J software was used for image analysis. Muscular ultrasound examinations were performed using the EPIQ CVx (Philips^®^, Andover, MA, USA), and the Vivid E95 (General Electric^®^, New York, NY, USA).

##### 2.2.2.2. Muscle strength

Muscle strength was evaluated using a handgrip test to assess upper limb strength, and a standing long broad jump to assess lower limb strength. Those tests are time-efficient, costless, validated as a general index of muscular fitness, and easily administered in clinical pediatric setting (39, 40). Measurements of upper limb strength from the handgrip test were based on protocols used in pediatrics (41). Grip notch was adjusted based on participant’s comfort. The participant squeezed the handgrip, with their right hand, for 3 to 4 s, as hard as they could. The procedure was repeated three times, with 30 s of rest between each trial. The maximum value of these three trials was reported. During standing long broad jump, the child stood behind jumping line, with feet together, and pushed off vigorously forward as far as possible (39). After the jump, participants were asked to stay still, with their two feet on the ground. If they lost their balance or touched any object, they were asked to repeat the jump. The distance between the jumping line and heel landing was measured. Among the two trials, the longest jump distance was reported.

### 2.3. Level of physical activity

The level of physical activity was evaluated using a waist-worn tri-axial accelerometer (ActiGraph GT3X, Pensacola, FL, USA) (42). The time spent at moderate and vigorous physical intensity was measured by the accelerometer. The device was assigned during the inclusion visit and the participants were instructed to always wear it at the waist for 14 days, except during sleep and water-based activities such as swimming or bathing. We chose an e-poch of 15 s (filtered acceleration signal over a user-defined time sampling interval) and non-wearing period was calculated by Choi’s algorithm (43). We fixed intensity thresholds (counts/min) using Romanzini’s equations for young adolescents (11–17 years old) (44), and Evenson’s equations, for children aged from 6 to 9 years old (45). Counts per minute thresholds were used to determine the level of moderate-to-vigorous physical activity (MVPA), the level of moderate physical activity and the level of vigorous physical activity. The three mean values were compared between LQTS and controls groups. A wearing period of 10 h per day for at least 4 days was necessary for the analysis (46).

### 2.4. Statistical analysis

Participant’s characteristics were presented using mean and standard deviation (SD) for continuous variables, and frequencies and proportions for categorical variables. After checking Q-Q plot, normality was violated. Therefore, we used Mann-Whitney test comparisons between groups. For categorical variable, we used Chi-square test. The effect size was estimated with Cohen’s d measure. The statistical significance was set at 0.05 and analyses were performed with R Studio software.

## 3. Results

### 3.1. Population

Twenty children with LQTS (12.7 ± 3.7 years old) and 20 healthy children (11.9 ± 2.4 years old) were included in the study. The two groups were similar in terms of age, gender, weight, height, and body mass index (Table 1). Children with LQTS were affected by the following genetic mutations, by descending order: KCNQ1 (*n* = 11, 55%), KCNH2 (*n* = 7, 35%), and KCNJ2 (*n* = 1, 5%). No mutation was found for one participant. Diagnosis modes were represented as follows: genetic diagnosis after known causal familial mutation (*n* = 12, 60% of which one prenatal diagnosis), incidental finding (*n* = 3, 15%), cardiac symptoms [*n* = 5, 25%, e.g., syncope (*n* = 2), ventricular extrasystole (*n* = 2), and bradycardia (*n* = 1)]. Most children with LQTS were prescribed beta-blockers (*n* = 18, 90%), of which nadolol (mean dose of 40 mg/m^2^/day) for 17 children and atenolol (0.5 mg/Kg/day) for one child. The mean QTc value was 458.3 ± 33.4 msec. None of LQTS had implantable cardioverter defibrillator.

**TABLE 1:** Demographic data and CPET values between LQTS and control groups.

	LQTS (*n* = 20)	Controls (*n* = 20)	*p*-value
Age (years)	12.7 ± 3.7	11.9 ± 2.4	0.46
Sex ratio	1.3 ± 0.5	1.5 ± 0.5	0.51
Weight (kg)	47.4 ± 15.9	44.4 ± 10.3	0.77
Height (cm)	151.8 ± 18.4	152.6 ± 13.9	0.82
BMI (kg/m^2^)	20.0 ± 2.9	18.8 ± 2.3	0.39
VAT (mL/Kg/min)	23.8 ± 5.1	28.8 ± 5.5	**0.007**
Percent-predicted VAT (%)	55.0 ± 10.8	67.8 ± 13.3	**0.002**
Peak heart rate (bpm)	147.9 ± 21.4	190.4 ± 9.3	**<0.001**
Percent-predicted peak heart rate (%)	75.6 ± 11.6	95.4 ± 6.4	**<0.001**
Maximum oxygen pulse (mL)	10.9 ± 2.7	9.2 ± 2.8	**0.04**
VE/VCO2 slope	32.5 ± 3.8	30.8 ± 5.2	0.23
Respiratory reserve (%)	28.8 ± 14.7	22.9 ± 16.2	0.28
Maximum power (watts)	126.5 ± 46.3	138.7 ± 59.1	0.61
Maximum RER	1.1 ± 0.1	1.2 ± 0.1	0.24

Results are expressed in mean ± SD; BMI, body mass index; LQTS, long QT syndrome; bpm, beat per minute; VAT, ventilatory anaerobic threshold; RER, respiratory exchange ratio. Significant *P*-values < 0.05 are marked in bold.

### 3.2. Cardiorespiratory fitness

**FIGURE 2:**
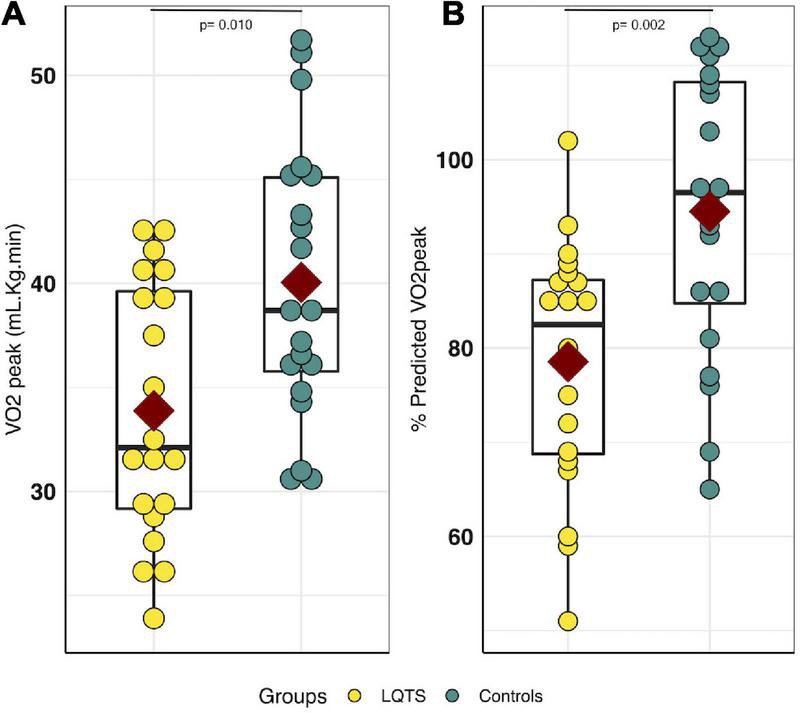
VO_2peak_ comparison between LQTS and control groups. **(A)** VO_2peak_ comparison. **(B)** Percent-predicted VO_2peak_ comparison. For each group, the dark red square represents the mean value for each group, the black boxplot represents the first, second and third quartiles [e.g., Q1, median, Q3], respectively. LQTS, long QT syndrome.

The mean VO_2peak_ in the LQTS group was lower than in the control group, in raw values (33.9 ± 6.2 mL/Kg/min *vs.* 40.1 ± 6.6 mL/Kg/min, respectively, *P* = 0.010, *d* = −0.96), as well as in percent-predicts (78.6% ± 13.1% *vs.* 94.5% ± 15.2%, respectively, *P* = 0.002, *d* = –1,12) (Figure 2). The proportion of children with an impaired cardiorespiratory fitness (e.g., percent-predicted VO2_peak_ < 80%) was 2.2 times higher in the LQTS than in the control group (45 *vs.* 20%). The VAT was lower in the LQTS group than in the control group, in raw values (23.8 ± 5.1 mL/Kg/min *vs.* 28.8 ± 5.5 mL/Kg/min, respectively, *P* = 0.007, *d* = –0.95), and in percent-predicts (55% ± 10.8% *vs*. 67.8% ± 13.3%, respectively, *P* = 0.002, *d* = –1,05). The proportion of children with an impaired VAT (e.g., percent-predicted VAT < 55%) was 2.5 times higher in the LQTS than in the control group (50 *vs.* 20%). Peak heart rate was lower in the LQTS group, and no significant difference was observed for maximal power and the main ventilatory parameters (VE/VCO2 slope, maximal RER). The mean VO_2_ in the LQTS group was not significantly different from the mean VO_2_ at the same heart rate (e.g., 140 bpm) in the control group (26.2 ± 4.35 mL/Kg/min *vs.* 27.4 ± 6.0 mL/Kg/min, respectively, *P* = 0.44, *d* = –0.23).

### 3.3. Muscular fitness

**FIGURE 3:**
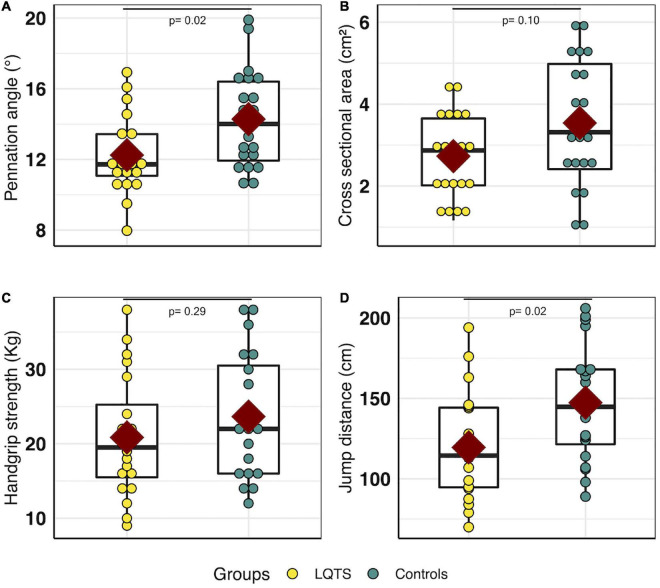
Muscle fitness comparison between LQTS and control groups. **(A)** Pennation angle (°). **(B)** Anatomical cross-sectional area (cm^2^). **(C)** Handgrip strength (kg). **(D)** Standing long broad jump distance (cm). For each group, the dark red square represents the mean value for each group, the black boxplot represents the first, second, and third quartiles [e.g., Q1, median, Q3], respectively. LQTS, long QT syndrome.

The pennation angle was significantly lower in the LQTS group than in controls (12.2° ± 2.4° *vs.* 14.3° ± 2.8°, respectively, *P* = 0.02, *d* = –0.8) (Figure 3A). The anatomical cross-sectional area was not significantly different between LQTS and control groups (2.7 ± 1.0 cm^2^ *vs.* 3.5 ± 1.6 cm^2^, respectively, *P* = 0.10, *d* = –0.6) (Figure 3B). The upper limb strength assessed by the handgrip test was not significantly different between the two groups (20.8 ± 8.2 Kg in LQTS and 23.6 ± 8.3 Kg in controls, *P* = 0.29) (Figure 3C). The lower limb strength assessed by the standing long broad jump distance was lower in the LQTS group than in controls (119.5 ± 33.2 cm *vs.* 147.3 ± 36.1 cm, respectively, *P* = 0.02, *d* = –0.8) (Figure 3D).

### 3.4. Level of physical activity

Valid wear time was appropriate in the LQTS group (mean 11 ± 3.6 days and 11.9 ± 1.8 h per day), as well as in the control group (mean 12.3 ± 2.2 days and 11.81 ± 1.5 h per day). Three participants were excluded from this analysis (two in LQTS group, one in control group) because the valid wear time period was not respected. MVPA was not significantly different between LQTS and control groups (36.9 ± 12.9 min/day *vs.* 41.5 ± 18.7 min/day, respectively, *P* = 0.66). The levels of physical activity were not significantly different between LQTS and control groups in terms of MVPA (36.9 ± 12.9 min/day *vs.* 41.5 ± 18.7 min/day, respectively, *P* = 0.66, *d* = –*0.2*), moderate physical activity (22.13 ± 12 min/day *vs.* 22.57 ± 11.16 min/day, respectively, *P* = 0.88, *d* = 0.03), and vigorous physical activity (14.31 ± 7.65 min/day *vs.* 19.97 ± 16.58 min/day, respectively, *P* = 0.46, *d* = 0.4). Two participants from the control group, and none in the LQTS group, complied with the WHO guidelines (e.g., ≥ 60 min per day of moderate-to-vigorous physical activity).

## 4. Discussion

In this prospective controlled study, two major components of physical fitness, e.g., cardiorespiratory and muscular fitness, were significantly impaired in children with LQTS, in comparison with healthy participants, despite similar levels of physical activity in both groups.

In terms of cardiorespiratory fitness, the lower peak VO2 values in the LQTS group may reflect chronotropic limitation induced by beta-blockers, which represents the standard treatment in children with LQTS. Indeed, the VO2 at the same heart rate was similar between LQTS and controls. During exercise, heart rate has a greater contribution to peak VO_2_ than stroke volume (47, 48). Nevertheless, the origin of the cardiorespiratory fitness limitation seemed multifactorial. Whereas peak VO_2_ remains the most common method of assessing aerobic fitness, our results also found that the VAT was impaired in half of children with LQTS, suggesting the existence of peripherical limiting factors to physical capacity in this population. Classically, cardiac adaptation at submaximal exercise, up to 50% of peak VO_2_, mostly relies on stroke volume increase, rather than on heart rate (49). Similarly, Bratt et al. found that beta-blockers did not reduce exercise capacity in adolescents with hypertrophic cardiomyopathy despite lower heart rate (50). Indeed, the VAT is more related to lactate metabolism than chronotropic adaptation (51). Physiologically, the lactate shuttle mechanism combines the production of lactate by active muscle fibers during exercise and the simultaneous consumption of lactate by adjacent fibers or distant sites as source of energy (52, 53). Previous studies have shown that lactate accumulation was mainly driven by oxidative capacity mechanism, e.g., oxidative enzyme, mitochondrial reticulum volume density (54), and capillary density. Therefore, the impaired VAT in children with LQTS may reflect some degree of altered oxidative mechanism and dysfunction of blood lactate shuttle mechanism.

In terms of muscular fitness, the lower strength muscle in lower limb may suggest the existence of neuro-muscular and/or glycolytic metabolism alteration in LQTS. Indeed, previous studies showed subclinical electromyographic alterations in adult with LQTS, but whether these abnormalities are neurogenic or myogenic remains unclear (55, 56). Pennation angle was significantly lower in children with LQTS, while anatomical cross-sectional area, although diminished, did not reach statistical significance. Considering that muscular adaptation to increase muscle strength after undergoing resistance training in children is mainly due to neuromuscular adaptation and coordination rather than hypertrophy (57), the same process may occur during muscle loss, reversely. Therefore, lower pennation angle and lower strength could result more from decreased muscle tension or poorer neuromuscular adaptation than from decreased muscle mass. The direct implication of beta-blockers on altered muscular fitness seems irrelevant, as they commonly have no effect on muscle excitability and fatiguability (58, 59). Muscular fitness assessment in clinical follow-up of children with chronic diseases may be of interest, however further studies are necessary to evaluate the underlying causal mechanism.

In this study, despite lower aerobic fitness, the level of physical activity assessed by accelerometer in children with LQTS was similar to that of healthy controls, as in previous studies using physical activity questionnaires (60). The weak association between objectively measured daily physical activity and aerobic fitness has been previously highlighted (61–64). Overall, no child with LQTS and only two healthy participants respected the WHO guidelines on physical activity (e.g., ≥ 60 min per day of MVPA). These results reflect the major public health issue of physical inactivity in children, whether they have a chronic disease or not (6). Previous studies observed that children with LQTS were commonly withdrawn from competitive sports, and as a precautionary principle, from many recreational sports activities (18, 23, 25). Considering that a cardiovascular training effect requires moderate-to-vigorous exercise intensity, the decrease in aerobic fitness observed in children with LQTS may reflect imposed activity restrictions. Yet, exercise-related events are exceptional in appropriately managed children with LQTS (65). Fortunately, the recent 2020 ESC guidelines on competitive sports participation in athletes with cardiovascular disease have taken an important step forward in promoting physical activity in patients with cardiac diseases, especially by introducing the concept of shared-decision making between patients and physicians (22). Nevertheless, the appropriate level of intensity and the type of physical activity for patients with LQTS need to be clarified, especially for pediatric patients. Therefore, it could be relevant to enroll patients with LQTS into cardiovascular rehabilitation programs, from pediatric age, as in congenital heart disease (31). Indeed, cardiovascular rehabilitation involves established core components, including exercise, patient education, psychosocial counseling, risk factor reduction and behavior modification, with a goal of optimizing patient’s quality of life (12, 31).

## 5. Study limitations

Some factors of cardiorespiratory fitness, such as nutrition, psychological factors, environment, or patients compliance to medical guidelines (19) have not been investigated in this study. Control participants were enrolled during a consultation at the hospital, and therefore may not be considered as healthy as if they were recruited from the general population. Some participants were enrolled during the COVID-19 pandemic lock-down, however, during this period (April 2021), subjects from both groups were evenly recruited and outdoor physical activities remained allowed within a 10 kilometer-perimeter. Because of the sample size study, no multivariate analysis could be performed, however, this pilot study led to the larger ongoing QUALIMYORYTHM trial, which will further explore the main determinants of physical components in children with inherited cardiac diseases (66).

## 6. Conclusion

In children with LQTS, cardiorespiratory fitness determined by peak oxygen uptake and ventilatory anaerobic threshold, and muscle fitness were lower than in healthy controls, despite similar levels of physical activity. The origin of this limitation seemed to be multifactorial, involving beta-blocker induced chronotropic limitation, physical and muscle deconditioning. Cardiopulmonary exercise testing and muscular evaluation may be of interest in pediatric LQTS follow-up and participate in promoting safe physical activity in this population.

## Data availability statement

The raw data supporting the conclusions of this article will be made available by the authors, without undue reservation.

## Ethics statement

This study was conducted in compliance with the Good Clinical Practices protocol and Declaration of Helsinki principles. It was approved by a drawn national Ethics Committee (CPP Sud-Est VI, 2020-A00411-38). Informed consent was obtained from all parents or legal guardians, and oral assent was obtained from all children. Written informed consent was obtained from the minor’s legal guardian for the publication of the potentially identifiable image (Figure 1). The study was registered on Clinicaltrials.gov (NCT04712136).

## Author contributions

LS, MA, AB, OW, and PA contributed to the study design and participants enrollment. AR, SM, MV, GD, VP, J-LP, and SG contributed to participants enrollment. LS, MA, and PA contributed to writing the manuscript. All authors approved the final version of the manuscript.
